# Biomarkers to Predict DMARDs Efficacy and Adverse Effect in Rheumatoid Arthritis

**DOI:** 10.3389/fimmu.2022.865267

**Published:** 2022-03-28

**Authors:** Kai Wei, Ping Jiang, Jianan Zhao, Yehua Jin, Runrun Zhang, Cen Chang, Lingxia Xu, Linshuai Xu, Yiming Shi, Shicheng Guo, Dongyi He

**Affiliations:** ^1^ Guanghua Clinical Medical College, Shanghai University of Traditional Chinese Medicine, Shanghai, China; ^2^ Department of Rheumatology, Guanghua Hospital Affiliated to Shanghai University of Traditional Chinese Medicine, Shanghai, China; ^3^ Arthritis Institute of Integrated Traditional and Western Medicine, Shanghai Chinese Medicine Research Institute, Shanghai, China; ^4^ The Second Affiliated Hospital of the Shandong University of Traditional Chinese Medicine, Jinan, China; ^5^ Center for Precision Medicine Research, Marshfield Clinic Research Institute, Marshfield, WI, United States; ^6^ Department of Medical Genetics, School of Medicine and Public Health, University of Wisconsin-Madison, Madison, WI, United States

**Keywords:** rheumatoid arthritis, therapy, biomarker, DMARDs, response, nonresponse

## Abstract

Rheumatoid arthritis (RA), one of the most common immune system diseases, mainly affects middle-aged and elderly individuals and has a serious impact on the quality of life of patients. Pain and disability caused by RA are significant symptoms negatively affecting patients, and they are especially seen when inappropriate treatment is administered. Effective therapeutic strategies have evolved over the past few decades, with many new disease-modifying antirheumatic drugs (DMARDs) being used in the clinic. Owing to the breakthrough in the treatment of RA, the symptoms of patients who could not be treated effectively in the past few years have been relieved. However, some patients complain about symptoms that have not been reported, implying that there are still some limitations in the RA treatment and evaluation system. In recent years, biomarkers, an effective means of diagnosing and evaluating the condition of patients with RA, have gradually been used in clinical practice to evaluate the therapeutic effect of RA, which is constantly being improved for accurate application of treatment in patients with RA. In this article, we summarize a series of biomarkers that may be helpful in evaluating the therapeutic effect and improving the efficiency of clinical treatment for RA. These efforts may also encourage researchers to devote more time and resources to the study and application of biomarkers, resulting in a new evaluation system that will reduce the inappropriate use of DMARDs, as well as patients’ physical pain and financial burden.

## Introduction

Rheumatoid arthritis (RA) is an autoimmune disease that can affect the structure of joints. RA patients struggle to respond to therapeutic strategies, and this may lead to remission. In the past decade, the marketing of many new drugs have provided more treatment possibilities for RA patients. There are several medications for the treatment of autoimmune diseases; however, standardized and accurate criteria for treatment must be included, which would enable doctors and patients to reduce unnecessary wasting of time and resources, alleviate patients’ suffering rapidly, and prevent joint deformities at an early stage (1). Clinicians and academics are relying on biomarkers that can assess the efficacy of pharmacological treatment to identify better pharmaceuticals and appropriate patient populations. Biomarkers, which are widely used in treatments, are biochemical indicators that can mark changes or possible changes in the structure or function of systems, organs, tissues, cells, and subcells. Biomarkers can be used to diagnose disease, determine disease stage, or evaluate the safety and efficacy of new drugs or therapies in target populations. Existing disease-modifying antirheumatic drugs (DMARDs) are primarily classified as conventional synthetic DMARDs (csDMARDs), biologic DMARDs (bDMARDs), or targeted synthetic DMARDs (tsDMARDs). Methotrexate (MTX) is a representative csDMARD, and its efficacy has always piqued the interest of researchers. As they are injections with a good efficacy and a wide range of applications, bDMARDs and their use have approached or exceeded the use of csDMARDs in many medical settings. In contrast, the effectiveness of tsDMARDs is measured primarily using a conventional clinical evaluation system. Owing to their late adoption in the clinic, targeted biomarkers are currently being investigated. The classical evaluation system of disease activity, including the disease activity scoring system (disease activity score in 28 joints, DAS28) recommended by the European Federation for the Prevention and Treatment of Rheumatism (European League Against Rheumatism, EULAR), clinical disease activity index (CDAI), and simplified disease activity index (SDAI), serves as a significant guide for judging the initial and post-medication efficacy of biomarkers in clinical practice. Despite this, disease progression or relapse is seen in many RA patients with normal C-reactive protein (CRP) and erythrocyte sedimentation rate (ESR) measurements or normal scores for other hematological indexes, which negatively affect doctors’ and patients’ confidence in treatment. We aimed to assess the relationship between potential biomarkers and DMARDs to determine the clinical applications of DMARDs with higher accuracy. The potential biomarkers associated with RA therapeutic drugs in existing studies are divided into two categories: drug-responsive and non-responsive biomarkers. The number and function of biomarkers that respond to DMARDs are undeniably dominant.

## Potential Biomarkers for the Response to Methotrexate Therapy

MTX, one of the most important csDMARDs, was originally developed as an antifolate anticancer drug and has since been used to treat RA. It is stated in the literature that, in the absence of contraindications, MTX is the first choice nonbiologic DMARD treatment for RA, and it is frequently prescribed as part of combination therapy with synthetic DMARDs, such as sulfasalazine and/or leflunomide and/or hydroxychloroquine (2). Therefore, the efficacy evaluation of MTX still warrants attention.

S100A8 (MRP8) and S100A9 (MRP14) are major leukocyte proteins known as damage-associated molecular patterns (DAMPs), and they are found at high concentrations in the synovial fluid of RA patients. As a DAMP protein, calprotectin (S100A8/S100A9 protein) primarily reflects neutrophil activity (3). Calprotectin is a substantial and independent predictor of erosion progression and response to treatment, particularly in individuals who have received effective biotherapy, where high baseline calprotectin levels signal potential erosion damage (4). Serum calprotectin levels have been postulated to be a possible measure of inflammatory rheumatism (3). In the treatment of RA, the S100A9 protein can predict the responsiveness of MTX/etanercept (MTX/ETA) as a putative biomarker for RA (5). EC de Moel et al. found high circulating calprotectin (S100A8/S100A9 protein) levels in patients with RA who relapsed within 12 months after phasing out antirheumatic drugs (6), which confirmed this conclusion. Calprotectin (MRP8/14) may be insensitive to placebo effects in relatively short-duration proof-of-concept studies and sensitive to agents receiving effective treatment. In recent years, studies on serum MRP8/14 levels as diagnostic biomarkers for systemic juvenile idiopathic arthritis (JIA) in children with chronic fever and therapeutic responses, including those to MTX, have made some achievements (7–9). The role of biomarkers in assessing the efficacy of MTX in adult patients with RA is still worth investigating. However, not all biomarkers can be used for both diagnostic and therapeutic purposes. The soluble receptor expressed on myeloid cell-1 (sTREM-1) was found to have no clinical value in predicting RA response to MTX, even though it may contribute to the prediction of early-stage RA (10).

Interleukins (ILs), which are important cytokines involved in immune cell interactions, play a significant role in the onset and progression of RA. Many biologics are based on studies analyzing ILs, but some researchers discovered that plasma interleukin-6 (IL-6) levels are significantly reduced during MTX treatment and that post-treatment, IL-6 levels are a strong predictor of radiographic progression (11). This may serve as an additional reference for MTX treatment.

Matrix metalloproteinase-3 (MMP-3) is a vital element in the destruction of bone and cartilage in RA and has been a hotspot of RA diagnostic biomarkers in recent years (12, 13). MMP-3 has also been shown to be useful for monitoring treatment of RA. Uemura et al. (14) measured the MMP-3 level in 206 outpatients RA over 4 months and also made continuous MMP-3 measurements in RA patients treated with MTX alone or in combination with infliximab (IFX). The MMP-3 level decreased gradually after 12 and 24 weeks of MTX treatment (14). Patients who responded favorably to MTX showed a greater decrease than those who did not. MMP-3 levels decreased considerably 6 weeks after IFX treatment and decreased sharply for the next 48 weeks, suggesting that a continuous measurement of MMP-3 may be valuable in evaluating the efficacy of MTX and IFX therapy (14).

CD39 is an exonucleoside enzyme that is highly expressed in regulatory T cells (Tregs) and is responsible for the production of adenosine (ADO), a crucial anti-inflammatory mediator of MTX action. Tregs (CD4^+^CD25^+^FoxP3^+^) are thought to play an important role in reducing RA efficacy. It was discovered that the higher the expression of CD39 in Tregs, the stronger its inhibitory ability (15). The high frequency of CD39^+^ and CD4^+^CD25^+^CD39^+^ Tregs in peripheral blood is associated with the response of RA to MTX, and this can be used as a potential biomarker to predict MTX response. In a mouse model of arthritis, CD39 blockers reversed the anti-arthritic effects of MTX therapy. MTX unresponsiveness in RA was associated with low expression of CD39 on Tregs and decreased inhibitory activity of these cells by reducing ADO production. As a result, detecting low CD39 mean fluorescence intensity (MFI) on Tregs using FACS on a small sample of whole peripheral blood represents a noninvasive, rapid, and convenient procedure for predicting MTX nonresponsiveness in RA patients with >99 percent confidence, and thus represents a valuable option for RA therapy (16). On the other hand, studies on the ADO deaminase gene polymorphism and baseline serum level of ADO deaminase have shown that they do not correlate with MTX response (17).

Polyglutamation appears to be required for the preservation of steady-state MTX concentrations and activity, implying that polyglutamate levels may be linked to treatment effectiveness. However, although the results of certain erythrocyte investigations in RA suggest that levels of polyglutamated MTX (especially long-chain polyglutamate) are linked to therapy response (18–22), this link has not been indicated in all investigations (23, 24). The pharmacokinetics of infliximab and the formation of ATI have been linked to MTX polyglutamates, according to research (25). This finding is particularly interesting in patients with RA who are resistant to cDMARDs and must transition to anti-TNF biologic therapy, such as IFX therapy. Anti-drug antibodies appear to be a driver of treatment efficacy decline, according to growing research; thus, reducing this risk is anticipated to help preserve treatment response (26).

In 205 MTX-treated patients with newly diagnosed RA, single nucleotide polymorphisms (SNPs) in genes associated with ADO release (AMPD1, ATIC, ITPA, MTR, and MTRR) were studied (27). The alleles AMPD1, ATIC, and ITPA were found to be strongly associated with the chance of a favorable reaction (defined as a disease activity score ≤2.4). The presence of these SNPs, along with an additional SNP, MTHFD1, was paired with clinical parameters such as baseline disease activity, sex, smoking status, and presence of rheumatoid factor to create a predictive metric for MTX response (28). A multicenter SNP investigation has shown several gene–gene interactions linked to MTX responsiveness. A genotype including specific SNPs in the ATIC, SLC19A1, and ITPA genes was linked to poor MTX response in two patient cohorts (29). In the third cohort, age, sex, and anti-citrullinated protein antibody (ACPA) status were all predictive of responsiveness; the genotype was only associated with MTX response in ACPA-positive older men (29). A different study revealed potential associations between MTX responsiveness and the presence of SNPs in the *GGH*, *ATIC*, and *SLC19A1* genes (30). The MTX response in early-stage RA is also associated with several genetic variants in CHST11, which encodes carbohydrate sulfotransferase 11 (31). Meta-analyses have been performed only on SNPs in two genes so far, and these showed that the 80G>A SNP (rs1051266) in SLC19A1 has a strong relationship with MTX efficacy (32).

A proposed biomarker for DMARD response is DNA methylation, which occurs when a methyl group is introduced into a cytosine-guanine (C-G) dinucleotide (CpG). Approximately 70–80% of all CpGs in the genome are methylated (33), and they typically cluster in gene promoter regions, forming CpG islands, which are typically hypomethylated in transcriptionally active genes (34). There is emerging evidence of an interrelationship between DNA methylation and inflammation in the regulation of immune pathways (35). The cytokine IL-6, for example, has been shown to promote DNMT1 expression, which is linked to DNA methylation in T cells (36–39). T-cell differentiation, activation, and migration are regulated by DNA methylation levels (38, 39), and T-cell activation causes demethylation of the IL-2 promoter, resulting in IL-2 production (40). This type of DNA methylation disorder may play a role in the development of RA. For example, Cribbs et al. (41) claimed that hypermethylation in the NFAT binding site of the CTLA-4 promoter region resulted in decreased CTLA-4 production, which was linked to impaired Treg activity in RA.

DNA methylation has the potential to serve as a biomarker for RA treatment response. A study examined DNA methylation in CD4+ T cells from patients with jejunoileal arthropathy (JIA). While 145 differentially methylated probes (DMPs) were found to be associated between cases and controls (false discovery rate adjusted P < 0.1), only 11 DMPs remained when 4 MTX-treated individuals were removed, suggesting that MTX may play a role in DNA methylation in CD4+ T cells in JIA patients (42). Two CpG sites (cg21040096 and cg09894276) revealed methylation alterations at 4 weeks related to a clinical EULAR response by 6 months, according to a study involving RA patients with a good (n = 34) or poor (n = 34) response to MTX. Changes in methylation for three differentially methylated locations were associated with alterations in tender joint counts, three with changes in swollen joint counts, and four with changes in the CRP level. Four of the twelve CpGs (cg23700278, cg27427581, cg04334751, and cg26764200) were shown to have repeated links in a separate dataset of samples from the Rheumatoid Arthritis Medication Study (43). DNA methylation can be utilized as a biomarker to evaluate RA therapy and needs to be studied further in the future.

## Potential Biomarkers for the Response to bDMARD Therapy

Tumor necrosis factor inhibitor (TNFi) therapies have shown excellent therapeutic results as bDMARDs in first-line treatment for RA. Despite this, there are still cases of insufficient TNFi response in clinical practice, with only about one-third of patients showing a strong response to TNFi (ACR70) (44). Therefore, it is a challenge to apply TNFi to an appropriate crowd of RA patients to achieve better treatment expectations.

MRP8/14, an endogenous TLR-4 receptor agonist derived from neutrophils and macrophages with tissue- or cell-specific expression, was previously thought to be a marker of acute inflammatory cell activation. Owing to the considerable decrease in the serum MRP8/14 level in RA patients following treatment, this measurement has been established as a powerful predictor of biotherapy response in patients with RA at baseline and could be used to assess response to treatment across diverse mechanisms of action (including adalimumab, IFX, and rituximab) (45). In the early stages of drug research, quantitative changes in serum myeloid-related protein (MRP8/14) levels can be utilized to predict the potential efficacy of novel antirheumatic medications (46). As a result, employing MRP8/14 as a biomarker may have positive implications for the personalization, as well as cost-effectiveness, of treatment in RA patients starting biological antirheumatic medication (45, 47). When treated with TNFi, serum amyloid A (SAA) was even more sensitive to disease activity than CRP, suggesting that it could be utilized as a marker to determine the disease activity after treatment that may help to judge the efficacy of treatment (48). Studies at the genetic level have also aided in the evaluation of the effectiveness of TNFi. Ferrero-Iglesias et al. (49) evaluated 14 SNPs as potential biomarkers related to TNFi responses. The *PTPRC*, *IL-10*, and *CHUK* genes were identified, with RS10919563 in PTPRC being the most relevant since its RA risk allele was linked to remission improvement. As a result, *PTPRC* is the most reproducible genetic biomarker for TNFi response, whereas IL-10 and CHUK replication is positive but weaker, indicating that more research and evidence are needed. A genome-wide significant SNP related to the *PDE3A-SLCO1C1* gene (rs3794271) was discovered in a meta-analysis of RA cohort data in Spain and Denmark (50). According to Ciechomska et al. (51), serum circulating miRNA-5196 can be employed as a potential biomarker to monitor TNFi response, especially during the early stage of RA. Since the change in miRNA-5196 expression was more significant, it outperformed CRP in predicting the clinical response to anti-TNF therapy. The effects of DNA methylation in the analysis of RA treatment were observed. Two differentially methylated locations (DMPs) corresponding to the *LRPAP1* gene were discovered in a study that evaluated DNA methylation signatures in whole blood from 36 good and 36 poor responders to etanercept (ETA), a fusion protein consisting of the extracellular ligand-binding domain of the 75kD receptor for tumor necrosis factor-α and the constant portion of human IgG1 (52). Meanwhile, *LRPAP1* methylation levels were associated with the genotype of the rs3468 variant (52). However, additional replication trials in independent sample collections are required to determine the biomarker’s value.

After 6 months of treatment, ETA, a TNFi, dramatically reduced the soluble IL-18 receptor complex in the serum, a potential diagnostic biomarker for RA, in 29 RA patients, suggesting that it might be used to assess the efficacy of ETA (53). Alleles associated with ETA treatment response were associated with CD84 gene expression and CD84 gene expression was associated with disease activity according to a genome-wide association study (GWAS) meta-analysis of RA patients. In European patients, an SNP (rs6427528) in the *CD84* gene is a biomarker of responsiveness to ETA (54).

IFX is a chimeric human-mouse monoclonal antibody that blocks TNF-α, which is a useful TNF antagonist. It is well-known that disintegrin, MMP, and thrombotic unit 5 (ADAMTS5) play a crucial role in cartilage aggregative protein breakdown. Tsuzaka et al. theorized that baseline ADAMTS5 mRNA levels may be used to predict IFX responsiveness in patients with RA. They also found that RA patients with high baseline *ADAMTS5* mRNA levels did not improve following IFX treatment, implying that IFX is unable to prevent aggrecan degradation by ADAMTS5 (55). In other words, medicines that suppress ADAMTS5 expression may be beneficial for improving the therapeutic efficacy of IFX from the standpoint of finding novel medications (55). Smoking is considered an important pathogenic factor that causes many diseases, including cancer. The role of smoking in immune diseases is becoming increasingly prominent. Hyrich K et al. (56) reported predictors of RA response to selective anti-TNF-α therapy and showed that current smoking (serum cotinine) was associated with lower IFX response rates, although this was significant only in a multivariable analysis. Similar results were not observed with other anti-TNF-α agents, including ETA. A study identified that the critical value of serum-soluble folate receptor β (SFRβ) was 8 ng/mL, and the effective response to TNFi was 100% specific. Despite the data being overly ideal, it is reasonable to conclude that high serum SFR levels can be used as a biomarker for the anti-TNF drug response (57). Osteoclasts secrete tartrate-resistant acid phosphatase 5B (TRACP-5B), which can be used as a clinically relevant marker of bone resorption. At baseline, TRACP-5B levels were associated with radiographic injury severity, disease duration, and painful joint counts. According to the study, the measurements of serum TRACP-5B in patients with RA can reflect clinical and radiological measures of disease activity, treatment with certain biologics, and the degree of response to treatment (58). After 12 and 24 weeks of treatment in our patients, the serum TRACP5b levels decreased progressively with IFX treatment, while the MTX group showed no change, indicating that serum TRACP5b levels may be clinically correlated with the effects of IFX treatment (58).

Adalimumab (ADA) coupled to monocyte membrane TNF from RA patients unexpectedly increased its expression and binding to TNF-RII expressed on Tregs, according to a previous study. As a consequence, ADA expanded functional Foxp3^+^ Tregs to suppress Th17 cells through an IL-2/STAT5-dependent mechanism (59). Based on this view, Nguyen D et al. (60) found that the expression of TNF on the monocyte membrane is regulated by the p38/IL-10 signaling pathway and that its level of expression can predict whether or not patients respond to ADA treatment. This finding could also be applied to other TNFi medications; however, this has yet to be validated. Furthermore, as previously stated, calprotectin (MRP8/MRP14, S100A8/A9) has a similar academic expectation value to that of CRP and that of ESR in the diagnosis and activity evaluation of RA, and It can be utilized to evaluate the effect of TNF-ADA in the treatment of RA patients (61). The research data of Koga et al. (62) confirmed that the detection of serum soluble urokinase plasminogen activator receptor (uPAR) at baseline and after treatment could be a predictive biomarker for evaluating the efficacy of TNFi ADA in patients with RA.

Aside from TNFi, numerous monoclonal antibodies (bDMARDs) are used in clinical practice. TNFi, as it is well-known, does not cover all patients who respond well. For unknown reasons, approximately 30–50% of RA patients respond negatively to bDMARDs, primarily TNFi. The recommended treatment strategy for these patients is to switch to another biological treatment (63). Nguyen et al. (64) identified cartilage oligomeric matrix protein (COMP) as a strong predictive biomarker of response to abatacept (ABA) therapy in RA patients who had failed in their first anti-TNF-α therapy.

Rituximab is a chimeric mouse/human immunoglobulin G1 (IgG1) monoclonal antibody to CD20 cell surface antigens expressed on B lymphocytes (65). Circulating mir-125b is an miRNA overexpressed in RA. The researchers reported that high levels of miR-125b at the onset of the disease were linked to an excellent medical response to rituximab treatment three months later, implying that serum mir-125b could be used as a biomarker for predicting rituximab response (66).

Tocilizumab (TCZ) is a humanized antibody against the IL-6 (anti-IL-6) receptor. The pretreatment RA serum IL-6 measurement may aid in estimating residual disease activity after TCZ treatment and in predicting TCZ responsiveness (67). In addition, in the process of TCZ treatment compared with DAS28-CRP, the multi-biomarker disease activity (MDBA) score response amplitude was relatively small, which may be owing to the influence of IL-6 on the MBDA score. As a result, in light of the available clinical data, MBDA scores obtained during TCZ treatment should be interpreted with caution (68). Studies on determining the appropriate dose of subcutaneous TCZ in patients with classic CRP as a measurement standard combined with efficacy and tolerance results have also been confirmed by Japanese scholars (69). In TCZ-treated RA patients, the serum leucine-rich α2-glycoprotein (LRG) level in those with active disease (CDAI > 2.8) was also significantly higher than that of those in remission, so this measurement may serve as a biomarker (70).

Mavrilimumab, a monoclonal antibody targeting the GM-CSF receptor, has been successfully tested in patients with RA (71). Subcutaneous injection of mavrilimumab is effective and well-tolerated, with few adverse reactions being reported, in patients with RA (72). Mortensen et al. (73) confirmed that citrullinated and MMP degraded vimentin fragments (VICM) are released by activated macrophages. Treatment with mavrilimumab significantly reduced VICM release and peptide-arginine deiminase-2 (PAD-2) gene expression in RA patients, suggesting that mavrilimumab can target macrophage activation and that VICM may be a new blood-based biomarker of anti-GM-CSF response.

Predictive biomarkers for drug therapy, particularly those of nonresponse, are critical for precise treatment and economic benefit in RA, as they can help avoid unnecessary waste of medical resources and reduce patient suffering. Perez-Guerreroe et al. (74) evaluated an elevated serum p-glycoprotein (p-GP) level as a risk factor for the failure of response to treatment with DMARDs in patients with RA. They suggest that p-GP levels can be used as a clinical tool to assess patients’ risk of DMARD failure (75). In addition, the detailed analysis of genes and identification of SNPs involved in drug resistance and sensitivity may help to predict drug response in patients with RA. According to their results, an elevated p-GP level has a sensitivity of 78% for detecting patients with therapeutic failure and confers a threefold greater risk of therapeutic failure (75). HLADRB1 ‘shared epitope’ alleles have been linked to a lack of response to MTX monotherapy (76). GWAS identified potential risk loci for poor MTX responses, including confirmation of previously identified associations with DHFR, FPGS, and TYMS genes (77). A study showed that serum high major vault protein (MVP) levels are associated with non-response to treatment (78).

In addition to the foregoing, the evaluation system composed of multiple biomarkers will, in theory, provide additional options to patients for the prediction of the inefficacy of DMARD drug treatment if they are properly coordinated. For example, Mellors et al. (79) developed a predictive classification algorithm that integrates clinical disease measures, whole-blood gene expression data, and disease-associated transcribed SNPs to identify those individuals who will not achieve an ACR50 improvement in disease activity in response to anti-TNF therapy. Similarly, the molecular signature response classifier (MSRC) developed by Strand et al. (80) could fundamentally shift treatment paradigms in RA by predicting nonresponse to TNFi, resulting in substantial improvements in treatment (**Table 1**).

**Table 1:** The Potential Biomarkers to Predict DMARDs Efficacy and Adverse Effect in RA.

Drugs Name		Biomarkers	Reference
**Response**			
**MTX**		Calprotectin(S100A8/A9)	(4, 5)
		Calprotectin (Serum MRP8/14)	(6–8)
		Plasma IL-6	(11)
		MMP-3	(14)
		CD39	(15, 16)
		Polyglutamation	(17–20)
		SNPs	(26, 28, 29)
		DNA methylation	(41, 42)
**TNFi**		Calprotectin (Serum MRP8/14)	(44–46)
		Serum Amyloid A	(47)
		SNPs	(48, 49)
		MiRNA-5196	(50)
		DNA methylation (LRPAP1)	(51)
**TNFi**	ETA	soluble interleukin-18 receptor complex	(52)
		SNP (rs6427528)	(53)
		Calprotectin (S100A9 protein)	(5)
	IFX	MMP-3	(14)
		ADAMTS5	(54)
		Smoking (Serum cotinine)	(55)
		Serum-soluble folate receptor β (sFRβ)	(56)
		TRACP-5b	(57)
	ADA	p38/IL-10 signaling pathway	(59)
		Calprotectin (S100 protein)	(60)
		soluble urokinase plasminogen activator receptor	(61)
**Abatacept(ABA)**		cartilage oligomeric matrix protein (COMP)	(63)
**Rituximab**		Serum miR-125b	(65)
**Tocilizumab (TCZ)**		Serum IL-6	(66)
		C reactive protein (CRP)	(68)
		Serum Leucine-rich α2 -glycoprotein (LRG)	(69)
**Mavrilimumab**		citrullinated and MMP degraded vimentin fragment (VICM)	(72)
**Non-response**			
**DMARDs**		p-glycoprotein (p-GP)	(73, 74)
**MTX**		HLA-DRB1 ‘shared epitope’ alleles	(75)
**RA treatment**		major vault protein (MVP)	(77)

## Discussion and Future Perspectives

**Figure 1:**
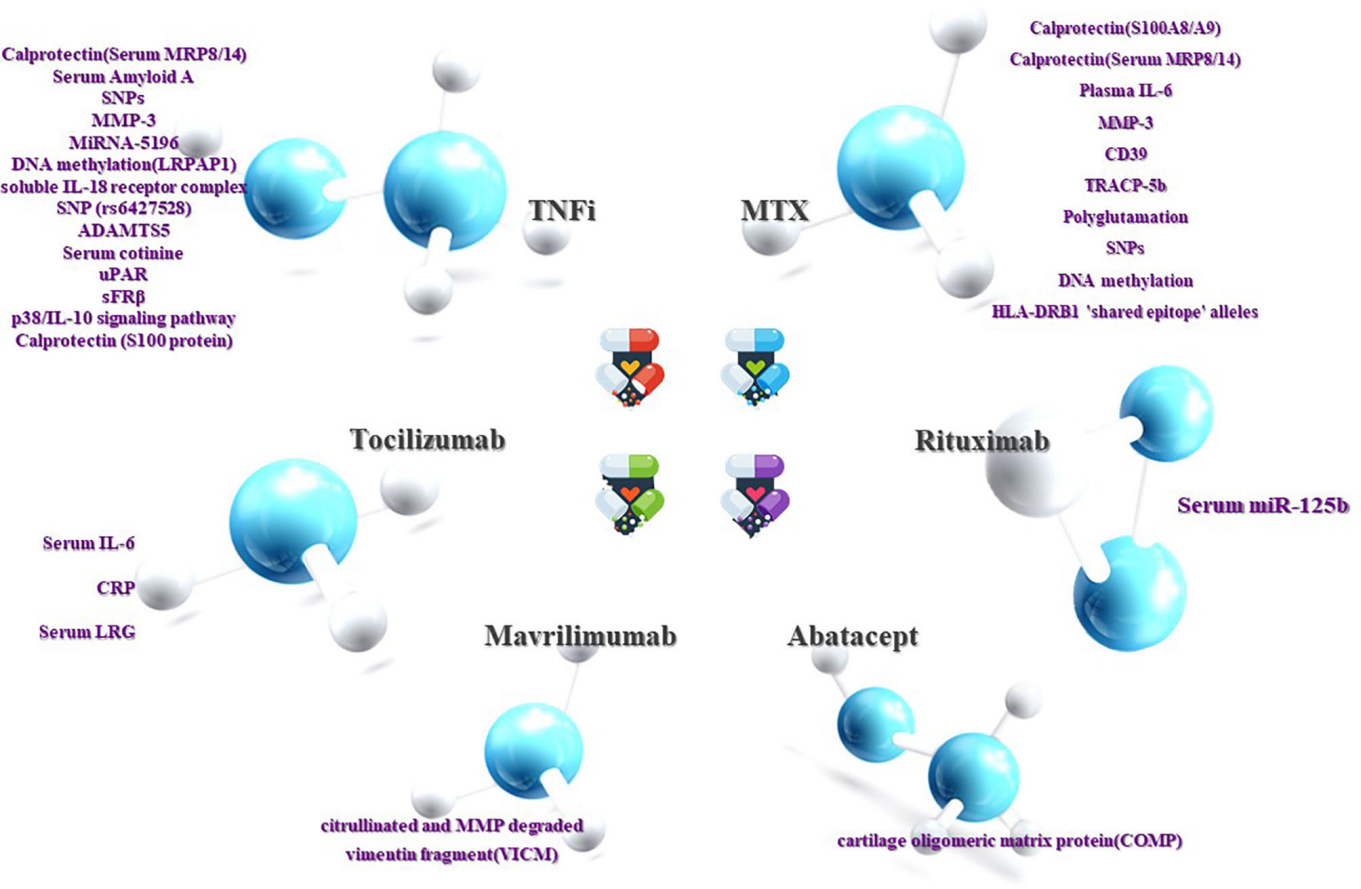
Prediction of RA efficacy by biomarkers. The ability to influence clinical decision-making is dependent on the response or non-response of RA patients to DMARDs drugs. Although current research on the treatment of non-responsive biomarkers is not as sufficient and accurate as the former, it can still be used as a supplement. Both are beneficial in clinical decision-making and complement each other.

There may be some limitations in our work, such as inaccurate keywords and the use of a limited number of databases. Moreover, owing to the time limit of our publication, some articles may not be read or cited, which may lead to incomplete conclusions. Despite this, researchers have discovered a large number of biomarkers with predictive value for RA treatment that is likely to be employed in clinics, and the fact that the feasibility of some biomarkers has been regarded as transcending the existing evaluation system is encouraging (**Figure 1**).

In addition to endogenous biomarkers, many scholars have explored the influence of environmental factors on the treatment of RA. Smoking is an environmental factor that has become important in the development and prognosis of RA. Maska et al. (81) observed a significant difference in the mean value of DAS28 scores between smokers and non-smokers between 48 and 102 weeks by studying serum cotinine levels. In contrast, Saevarsdottir S et al. (82) observed a significantly lower rate of response to RA therapy in current smokers undergoing MTX or TNF-inhibited therapy compared to those who reported a never-smoking status, although the primary outcome measured response to therapy only after 3 months of therapy. Above all, these could lead to more accurate use of targets and prognostic indicators for rituximab in clinical trials.

This review article lists biomarkers that can help predict the efficacy and adverse reactions of DMARDs, all of which come from recent studies, a very small number of them have been used clinically, and most of them still need more research data to support them. Some assumptions have been validated in animal models or *in vitro* studies, which are gratifying findings, but biomarkers as a predictor of efficacy cannot stop there. Some studies based on human clinical samples seem to be more reliable, but they still need a larger number of samples and are constantly revised in later applications to improve accuracy of prediction. In recent years, there are many research hotspots related to RA treatment emerge one after another, and they are expected to become new predictive biomarkers. The p53 tumor suppressor protein plays an integral role in apoptosis. Changes in peripheral lymphocyte (PL) apoptosis may be linked to RA. In the past few years, Moodley et al. (83) have suggested that the p53 codon 72 genotype of the tumor suppressor gene does not affect PL apoptosis or mitochondrial depolarization and that it is not associated with clinical disease markers of RA. In recent RA studies, p53 has been re-mentioned as an efficient marker in the progression and interpretation of RA disease activity (84–87). Following its discovery, p53 is expected to become a biomarker for assessing the efficacy of RA treatment.

In addition, many researchers combine multiple biomarkers, which may enhance the predictive value of biomarker model for DMARDs response. For example, according to Nguyen et al., a multivariate model integrating three biomarkers (prealbumin, platelet factor 4, and S100A12) accurately predicted the response to TNFi in RA patients and has the potential in serving as tailored treatment in daily practice (88). This provides us with a hypothesis for our study: taking into account a wide range of factors could improve prediction accuracy.

Standard treatment regimens can no longer meet the requirements of individuals for the effectiveness of RA treatment, and known pathogenesis leads researchers to explore more accurate individualized treatment options (89), although not all of these efforts have positive results. For example, Smith et al. (90) attempted to explore the predictive value of CD11c expression in response to ADA and ETA, but the results showed that CD11c expression was not associated with TNFi biologic response in whole blood samples of RA patients before treatment. At present, our understanding of genetic markers is not reproducible. While GWASs have identified over 100 SNPs associated with RA susceptibility (91), this outcome has not been observed in genetic studies on treatment response (92).

Reviewing the work of researchers, it is evident that the academic community has tried to apply potential biomarkers in the evaluation of RA complications and efficacy, and some targeted, sensitive, and specific biomarkers have been identified. With further research, accurate and reliable biomarkers may be identified based on existing potential biomarkers, forming an effective evaluation system for drug efficacy and facilitating future drug development and clinical disease treatment practice. The application of biomarkers in drug efficacy has broadened their application scope, which may not only compensate for the traditional evaluation system’s inability to parallel real-world treatment feedback from patients in seronegative cases but also play an important role in guiding clinical precision and individualized delivery of traditional drugs and new preparations.

## Author Contributions

KW designed and wrote the manuscript including a figure by a critical discussion with PJ. SG, and DH revised the manuscript. All authors contributed to the final manuscript.

## Funding

This work was funded by the National Natural Science Funds of China (82074234 and 82004166); Shanghai Chinese Medicine Development Office, National Administration of Traditional Chinese Medicine, Regional Chinese Medicine (Specialist) Diagnosis and Treatment Center Construction Project-Rheumatology; State Administration of Traditional Chinese Medicine, National TCM Evidence-Based Medicine Research and Construction Project, Basic TCM Evidence-Based Capacity Development Program; Shanghai Municipal Health Commission, East China Region based Chinese and Western Medicine Joint Disease Specialist Alliance.

## Conflict of Interest

The authors declare that the research was conducted in the absence of any commercial or financial relationships that could be construed as a potential conflict of interest.

## Publisher’s Note

All claims expressed in this article are solely those of the authors and do not necessarily represent those of their affiliated organizations, or those of the publisher, the editors and the reviewers. Any product that may be evaluated in this article, or claim that may be made by its manufacturer, is not guaranteed or endorsed by the publisher.
